# Supplementary material for: Optimization of Maduramicin Ammonium-Loaded Nanostructured Lipid Carriers Using Box–Behnken Design for Enhanced Anticoccidial Effect against Eimeria tenella in Broiler Chickens
Source: Pharmaceutics. 2022 Jun 23;14(7):1330. doi: 10.3390/pharmaceutics14071330 (PMC9323563; doi:10.3390/pharmaceutics14071330)
Supplement: Supplementary file 1 [file pharmaceutics-14-01330-s001.zip › pharmaceutics-1745201-supplementary.pdf]

pharmaceutics

Supplementary Materials: Optimization of Maduramicin Ammonium Loaded Nanostructured Lipid Carriers Using Box-Behnken Design for Enhanced Anticoccidial Effect against *Eimeria tenella* in Broiler Chickens

Yan Zhang [1,†], Runan Zuo [1,†], Xinhao Song [1], Jiahao Gong [1], Junqi Wang [1], Mengjuan Lin [1], Fengzhu Yang [1], Xingxing Cheng [1], Xiuge Gao [1], Lin Peng [1], Hui Ji [1], Xia Chen [2], Shanxiang Jiang [1], Dawei Guo [1*]

[1] Engineering Center of Innovative Veterinary Drugs, Center for Veterinary Drug Research and Evaluation, College of Veterinary Medicine, Nanjing Agricultural University, 1 Weigang, Nanjing 210095, PR China

[2] College of Animal Science and Technolog, Nanjing Agricultural University, 1 Weigang, Nanjing 210095, PR China

* Correspondence: gdawei0123@njau.edu.cn

† These authors contributed equally to this work.

Figure S1. The structure of stearic acid under light microscope.

Figure S2. The mixture of stearic acid and MAD under light microscope.

Figure S3. Relative weight gain of chickens in each group.

Figure S4. Survival rate of chickens in each group.

Figure S5. Cecal lesion score of chickens in each group. Compared to the uninfected-untreated group (Group 1), cecal lesions of chickens in infected-untreated group (Group 2), MAD-premix group (Group 3) and low-dose group of MAD–NLCs (Group 4) were extremely significantly different. ** $P < 0.01$.

Figure S6. Oocyst value of chickens in each group. Compared to the uninfected-untreated group (Group 1), oocyst values of chickens in infected-untreated group (Group 2), MAD-premix group (Group 3) and low-dose group of MAD–NLCs (Group 4) were extremely significantly different. ** $P < 0.01$.

Table S1. Screening of emulsifiers for mixed lipids.

Table S2. ANOVA of HD model.

Table S3. ANOVA of ZP model.

Table S4. Predicted and observed values for the optimization MAD-NLCs.

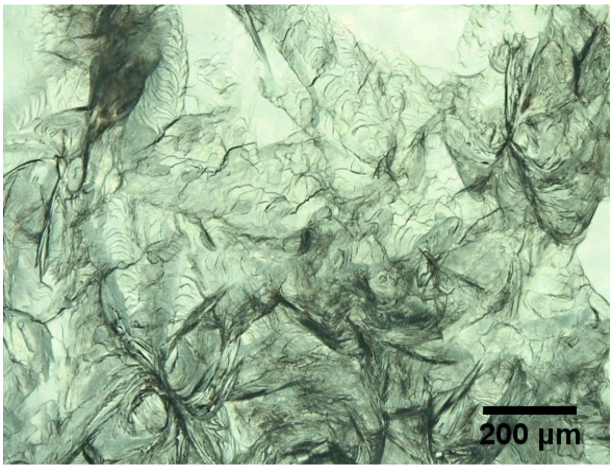

Figure S1. The structure of stearic acid under light microscope.

Publisher's Note: MDPI stays neutral with regard to jurisdictional claims in published maps and institutional affiliations.

Copyright: © 2022 by the authors. Submitted for possible open access publication under the terms and conditions of the Creative Commons Attribution (CC BY) license (https://creativecommons.org/licenses/by/4.0/).

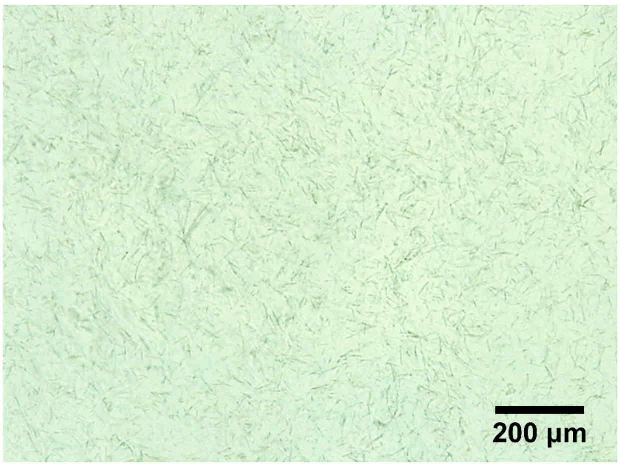

Figure S2. The mixture of stearic acid and MAD under light microscope.

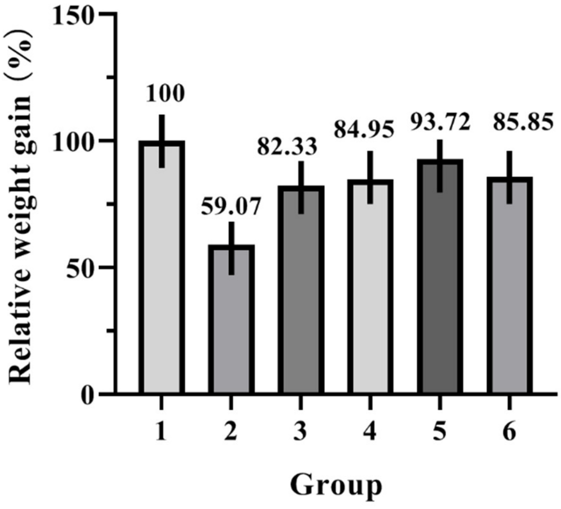

Figure S3. Relative weight gain of chickens in each group.

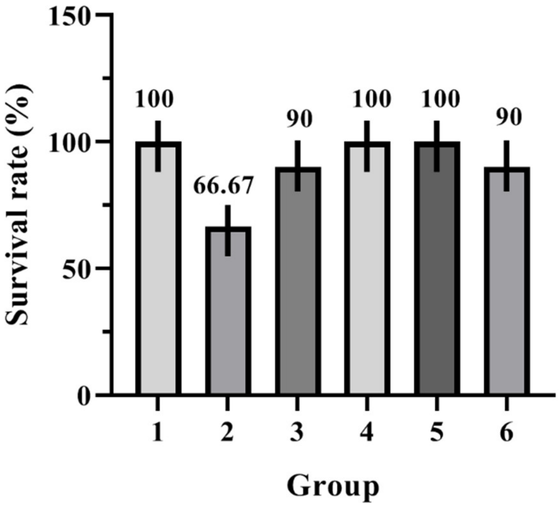

Figure S4. Survival rates of chickens in each group.

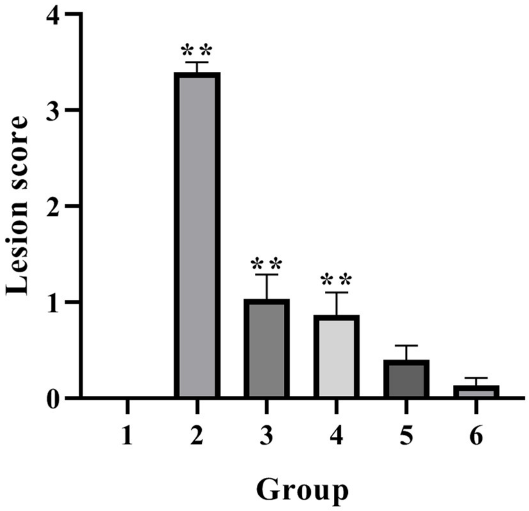

Figure S5. Cecal lesion scores of chickens in each group. Compared to the uninfected-untreated group (Group 1), cecal lesions of chickens in infected-untreated group (Group 2), MAD-premix group (Group 3) and low-dose group of MAD–NLCs (Group 4) were extremely significantly different. ** $P < 0.01$.

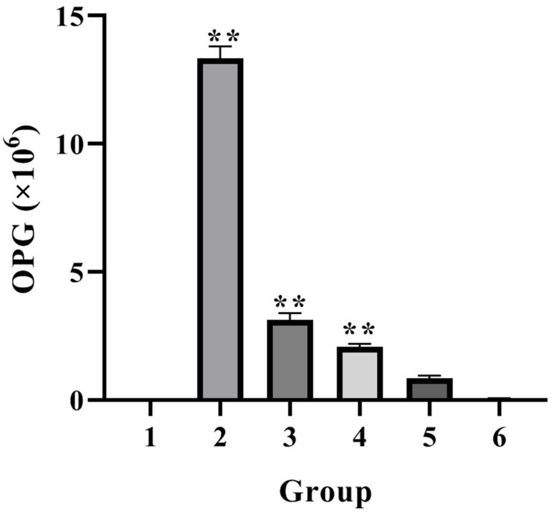

Figure S6. Oocyst values of chickens in each group. Compared to the uninfected-untreated group (Group 1), oocyst values of chickens in infected-untreated group (Group 2), MAD-premix group (Group 3) and low-dose group of MAD–NLCs (Group 4) were extremely significantly different. ** $P < 0.01$.

Table S1. Screening of emulsifiers for mixed lipids.

Emulsifier	Emulsified state		Precipitate
	Dispersion	Fluidity	
T20	+++	+++	-
T60	++	+++	-
T80	+++	+++	-
S80	-	+	+
P407	-	++	+
P188	-	++	+
T80 + P188	+++	+++	-
T80 + P407	++	++	-
T20 + P188	++	++	-
T20 + P407	++	++	-

Note: "+" indicated the occurrence degree of this phenomenon.
"-" meant no such phenomenon.

Table S2. ANOVA of HD model.

Variance sources	Sum of squares	DF	Mean square	F-value	P-value
Model	68950.07	9	7661.12	50.15	< 0.0001**
A	55012.44	1	55012.44	360.10	< 0.0001**
B	4227.40	1	4227.40	27.67	0.0012**
C	3515.41	1	3515.41	23.01	0.0020**
AB	4907.00	1	4907.00	32.12	0.0008**
AC	597.80	1	597.80	3.91	0.0884
BC	11.56	1	11.56	0.076	0.7912
A2	565.10	1	565.10	3.70	0.0959
B2	64.37	1	64.37	0.42	0.5370
C2	48.39	1	48.39	0.32	0.5911
Residual	1069.38	7	152.77		
Lack of fit	322.31	3	107.44	0.58	0.6611
Pure error	747.07	4	186.77		
Cor total	70019.45	16			
R^2	0.9847				
Adj-R^2	0.9651				
Pre-R^2	0.9097				
Adeq. pre.	26.346				
CV%	3.61				

Note: * meant $P < 0.05$, significant; ** meant $P < 0.01$, highly significant.

Table S3. ANOVA of ZP model.

Variance sources	Sum of squares	DF	Mean square	F-value	P-value
Model	240.47	9	26.72	25.24	0.0002**
A	15.40	1	15.40	14.55	0.0066**
B	14.58	1	14.58	13.77	0.0075**
C	2.53	1	2.53	2.39	0.1660
AB	11.56	1	11.56	10.92	0.0130*
AC	0.022	1	0.022	0.021	0.8882
BC	0.36	1	0.36	0.34	0.5781
A^2	45.78	1	45.78	43.25	0.0003**
B^2	19.96	1	19.96	18.86	0.0034**
C^2	129.58	1	129.58	122.40	< 0.0001**
Residual	7.41	7	1.06		
Lack of fit	0.50	3	0.17	0.097	0.9577
Pure error	6.91	4	1.73		
Cor total	247.88	16			
R^2	0.9701				
Adj-R^2	0.9317				
Pre-R^2	0.9240				
Adeq.pre.	17.789				
CV%	3.03				

Note: * meant P < 0.05, significant; ** meant P < 0.01, extremely significant.

Table S4. Predicted and observed values for the optimization MAD-NLCs.

Response	Predicted values	Observed values	Error (%)
SL (%)	10%	10%	-
EL (%)	30.58%	31%	-
ML (%)	30.00%	30%	-
HD (nm)	223.50	214.1±11.90	4.21%
ZP (mV)	-41.50	-42.8±1.05	-3.13%
